# Retraction to ‘Hedgehog pathway permissive conditions allow generation of immortal cell lines from granule cells derived from cancerous and non-cancerous cerebellum’

**DOI:** 10.1098/rsob.200136

**Published:** 2020-06-10

**Authors:** Constantin Heil

*Open Biol.* **9**, 180145. (Published Online 31 January 2019) (doi:10.1098/rsob.180145)

The publisher has retracted this article from *Open Biology* in agreement with the Editor-in-Chief because the author was not granted permission (implicit or explicit) to publish the data used to validate the study. The author of the article made use of data that he previously used as fulfilment of his PhD degree at The Department of Molecular Medicine of University La Sapienza Rome, where the work had been partially conducted and funded in 2011–2014, and submitted the paper without permission from his laboratory supervisor, Prof. Giuseppe Giannini. The Editor is satisfied that the veracity of the content and key conclusion of the article is unaffected. Following determination by the university's Research Integrity Office, the article in question is being retracted.

Dr Constantin Heil

Prof. Jon Pines FRS Editor-in-Chief, *Open Biology*

